# Manipulations of the Response-Stimulus Intervals as a Factor Inducing Controlled Amount of Reaction Time Intra-Individual Variability

**DOI:** 10.3390/brainsci11050669

**Published:** 2021-05-20

**Authors:** Paweł Krukow, Małgorzata Plechawska-Wójcik, Arkadiusz Podkowiński

**Affiliations:** 1 Department of Clinical Neuropsychiatry, Medical University of Lublin, ul. Głuska 1, 20-439 Lublin, Poland; 2 Institute of Computer Science, Lublin University of Technology, ul. Nadbystrzycka 38A, 20-618 Lublin, Poland; m.plechawska@pollub.pl; 3 Specialized Neurosurgical Practice, ul. Hirszfelda 3/9, 20-092 Lublin, Poland; apodkowinski@wp.pl

**Keywords:** intra-individual variability, cognitive speed, response-stimulus interval, default mode network

## Abstract

Aggrandized fluctuations in the series of reaction times (RTs) are a very sensitive marker of neurocognitive disorders present in neuropsychiatric populations, pathological ageing and in patients with acquired brain injury. Even though it was documented that processing inconsistency founds a background of higher-order cognitive functions disturbances, there is a vast heterogeneity regarding types of task used to compute RT-related variability, which impedes determining the relationship between elementary and more complex cognitive processes. Considering the above, our goal was to develop a relatively new assessment method based on a simple reaction time paradigm, conducive to eliciting a controlled range of intra-individual variability. It was hypothesized that performance variability might be induced by manipulation of response-stimulus interval’s length and regularity. In order to verify this hypothesis, a group of 107 healthy students was tested using a series of digitalized tasks and their results were analyzed using parametric and ex-Gaussian statistics of RTs distributional markers. In general, these analyses proved that intra-individual variability might be evoked by a given type of response-stimulus interval manipulation even when it is applied to the simple reaction time task. Collected outcomes were discussed with reference to neuroscientific concepts of attentional resources and functional neural networks.

## 1. Introduction

The studies analysing relatively simple, yet speed-dependent elementary cognitive processes, most often using experiments where response time (or reaction time: RT) is the primary indicator of performance are among the most classical strands of psychology and neurosciences [1]. In the majority of chronometric research, the main outcome of applied tasks is a series of RTs enabling computation of mean RT (mRT), but additionally, a set of consecutive RTs is often characterized by more or less pronounced fluctuations giving insight into the scope of trial-to-trial intra-individual variability (IIV) [2,3]. In the context of RT-studies, IIV or processing consistency refers to unintentional changes in performance efficiency, e.g., its speed, which are not secondary to modification in experimental conditions, but rather reflects intrinsic oscillations in attention and vigilance [4]. Many studies confirmed that IIV is indicative of fluid intelligence [5,6,7], developmental inquires showed that IIV is a valid marker of white matter maturation and cognitive ageing [8,9,10], and generally neuronal health and brain integrity [11]. 

Precise assessment and explanation of processing inconsistency origin is also an important aspect of research on patients with neuropsychiatric disorders of various aetiology. It was shown that in major psychotic and affective disorders [12,13,14], as well, as in individuals with acquired brain injuries [15], processing variability is a valid measure of overall cognitive decline, moreover, RT-related inconsistency has been observed even in cognitively high-functioning schizophrenia patients [16]. There is also a growing body of literature demonstrating that IIV measures, similarly to complex and extensive neuropsychological batteries, are very sensitive indicators of subtle neurocognitive disorders present in selected neurological [17], psychiatric [18] and somatic diseases [19]. IIV may also be used as a valid parameter in predictions regarding risk of dementia onset [20,21]. Of note, increased IIV explained a substantial part of processing speed deficit in schizophrenia and bipolar disorder [22,23], what is of special importance considering that slowdown is not only one of the most pronounced neuropsychological impairment in these groups, but also explain differences between patients and controls regarding all other cognitive dimensions [24,25,26]. 

Despite this compelling evidence of a link between regularity of time-limited performance and overall measures of cognitive capacity, studies in neuropsychiatric populations carried out so far, are characterized by vast heterogeneity regarding methods used to assess consistency, and first of all, the usage of tasks evaluating relatively complex cognitive dimensions, such as decision making and executive functions [12,27]. In fact, IIV is often calculated, somewhat incidentally, on the basis of data originating from the tests which were not purposely designed to assess the performance consistency [28,29]. In clinical groups in which neuropsychological impairments concerns many domains and reach various levels of severity, application of task assessing higher-order cognitive functions to analyze the level of temporal consistency might in fact impede precise conclusion whether performance instability is an effect of disorder affecting given modular function (e.g., resistance to interference), or does it represent disturbances in elementary cognitive processes, such as establishing stimulus—reaction association, which would be present regardless of the type of evaluated function. So, it seems justified to create the method allowing for evaluation of RT-performance consistency without applying complex tests, but with the most basic stimulus-response association tasks, especially if one aim to assess clinical populations regarding relationships between elementary processing stability and more complex cognitive dimensions (e.g., executive functions) [30,31]. Therefore, we attempted to construct the method containing almost the simplest possible type of cognitive task, but, with a structure conducive to reveal intrinsically-conditioned tendency to perform variably. We assumed that this effect might be achieved by applying an extended and irregular length of the response-stimulus intervals (RSI), even, when it is implemented to a simple reaction-time task (SRT). According to the concept of the supervisory attentional system [32,33], when the subject performs simple task in which the main stimuli are separated by long intervals of variable length, then for a testee, the goal-directed behaviour must be maintained internally, what eventually overloads mechanisms retaining the optimal level of preparedness, causing periodic decrements in attentional efficiency [34]. In the case of irregular RSIs, a testee cannot elaborate an anticipatory response, as it is possible with regular RSIs, which additionally burdens the possibilities to sustain attention [2]. Independently of these studies, Killen [35] provided further theoretical rationale suggesting that extended RSIs will increase performance inconsistency. Since paying attention is a very energy-consuming process, the necessity to stay focused for prolonged time intervals especially in the low-paced task, without external stimulation, will exhaust energetic resources and lead to states of inattention and mind wandering. As an example, Killen pointed out at Leth-Steensen et al. study [36] on individuals with attention-deficit/hyperactive disorder, in which authors applied four-choice RT-tasks with RSIs of 2, 4 and 8 s. length and demonstrated that probability of inattention increased from 22% for 2 s. foreperiod to around 60% for 8 s. foreperiod. This seems to additionally support the assumption that slow-paced tasks with at least few RSIs lasting several seconds (e.g., 8) might be specifically sensitive to inattention-related performance variability [37]. 

Considering the above, our study had two main goals: to develop and present relatively new, simple and convenient assessment method which deliberately elicit variable performance and to verify whether the gradual increase of the RSI’s length and irregularity will generate a gradual increment of RT-related variability. Specifically, we want to determine whether, with the gradual lengthening and increase in RSI irregularities, the RT distributions will be more skewed and above all, whether the hypothesised IIV increment will also be observed if the correction for mRT will be taken into account. The construction of the tasks was based on the theoretical premises characterized above. While developing the method, we had in mind, that it is intended to use in studies including patients with serious neuropsychiatric disorders (e.g., after traumatic brain injury, with neurodegenerative diseases and psychoses), therefore the tasks are simple and relatively short, the stimulus material is perceptively simplified. In addition, we were aware of the implications from previous studies on dynamics of the information processing in neuropsychiatric patients, such as the need to control for the manual speed and variability and disturbances in initiating consistent performance [22]. It was presumed that the significant differences between any clinical and control group regarding intra-individual variability for the SRT task implies disruptions of elementary cognitive processes, such as establishing a stimulus-response associations, and to possibly least extent deficits in higher-order, complex cognitive domains. We also assumed that by designing several tasks with increasing levels of RSI length and irregularity, in further research it will be possible to compare clinical and control groups not only with regard to results of individual test, but also in the matter of the degree of the expected increase in cognitive instability, with an increasing level of RSI complexity. Of note, the last subtest with the longest and most variable RSI is considered as the main task eliciting IIV. 

The current study encompasses a sample of intellectually active healthy young adults because it was hypothesized that if the expected effect of RSI length and regularity on the level of IIV will occur in such a group, then it is very probable that it will be evident in clinical samples containing patients prone to process inconsistently. Since it is highly probable that increasing RSI complexity will elongate mRT [38,39], we planned to focus on such indicators of performance variability as selected ex-Gaussian parameters and coefficient of variation (CoV), which are separated from the mRT or include a correction for mRT. Considering previous findings [40] demonstrating that there are probable sex differences regarding performance speed and variability, at the end of analysis, the outcomes of all tasks will be compared between male and female participants. 

## 2. Materials and Methods

### 2.1. Subjects

The target research group included right-handed healthy students from local universities, aged 19–25, about equal number of men and women. Students represented technical, medical and social faculties and were invited during the lectures. Due to the fact that collected results might be used as a normative data for healthy controls compared, for example with patients, but also non-clinical at-risk samples, additional participation rules were taken into account. However, it should be remembered that possible application of data collected from this group of healthy controls will probably require an adjustment for the level of education. Therefore, after gathering 135 volunteers meeting inclusion principles, each of them were interviewed in terms of more specific exclusion criteria. The interview was conducted by a psychologist and concerned the following issues: past or present history of any neurological disorders, including epilepsy, concussion and other forms of TBI, consultation with a neurologist for any reason and taking medications prescribed by a neurologist, past or present history of any psychiatric disorders, consultation with a psychiatrist for any reason and taking medications prescribed by a psychiatrist, a history of any neurodevelopmental problems, including specific language and learning disorders, problems with promotion in primary or secondary school, family burden with neuropsychiatric diseases, including first-degree relatives, with special interest in psychoses, major mood disorders, suicide deaths and early-onset dementias, current taking of any drugs or other substances affecting psychological state, such as alcohol, stimulants, energetics, anxiolytics. In case when volunteer revealed the existence of any of the above feature or experience she/he was not engaged in the main study. Participants were also asked to refrain from consuming coffee or nicotine at least one hour before tests administration. The test took place between 10 a.m. and 3 p.m., and was carried out individually in a well-lit and quiet room. 

### 2.2. RT-Related Cognitive Tasks

The original method developed to answer the research problems was a cognitive mini-battery called “RT-dynamics” containing four separated digital tasks presented as a tablet application. The applications were programmed using C# language in Xamarin framework, which is an extension of. NET platform that enables to build mobile solutions for different platforms. Our application is dedicated for Android devices. All tasks were preceded by a displayed instruction that the subject read on his own, without any time limit. During reading the instructions, the subjects could ask questions about the rules of task performance. After getting acknowledged with the instructions, the respondents proceeded to a probationary trial. Any errors made during probationary trial prevented the transition to the main task. With such errors, the probationary trial had to be performed again, but no more than twice. All tasks were performed in exactly the same order. The first task (*FT*) was a typical finger-tapping test administered to measure manual speed. It contained a black dot about 1.5 cm in diameter placed in the center of a white background, which was also a touchscreen. The task was to tap the dot as quickly as possible with an index finger of the right hand until the test will finish. A probationary trial consisted of tapping continuously through 10 s or tapping 20 times. In the main task, a subject had to tap the dot 100 times and thus giving 100 measures covering the time between all pairs of consecutive taps. Based on these 100 time measures it was possible to compute indicators of manual speed and variability, such as M, iSD and CoV. These psychometric measures of manual performance were later subtracted from the psychometric parameters describing the RT-related performance of cognitive tasks, thus enabling to control for the possible impact of motoric aspects on measures of cognitive speed and variability. Subjects were reminded that they would have to perform further tests with their index finger. Subsequent cognitive tests were based on the SRT paradigm, the task was to tap the black dot as quickly as possible when it appears on the screen. The black dot was about 1.5 cm in diameter, and in all tests, it was the stimulus and a reaction button. All RTs from these tasks cover the interval between the stimulus display and its tapping. Instructions were generally the same in all three tasks, subjects had to tap the dot as quickly as it will appear on the screen and generally perform the task as fast as possible until it will finish and avoid errors such as touching the screen before the onset of the stimulus or tapping parts of the screen other than the black dot. The dot was displayed until it was touched. Individual tasks differed only in the length and regularity of the RSI. In the *T*1 task the RSI was short and fixed, the stimulus appeared 250 ms after the previous reaction. RSI in the next task (*T*2) ranged between 250 and 2000 ms. The exact length of each interval was randomly assigned by the computer program, but 2000 ms RSIs had to be implemented in 20% of all stimuli displays and total length of all successive RSIs within given task was always the same. The intervals between successive presentations were different to prevent the development of an anticipatory response. The last task (*T*3) was constructed very similarly to the previous one, but in this case, the RSIs ranged between 2000 to 8000 ms. Again, the length of each interval was randomly assigned by the computer program, but 8000 ms RSIs had to be implemented in 20% of all stimuli displays. None of the successive intervals had the same length. The so-called commission errors were recorded when a subject tapped the touchscreen before stimulus onset or tapped other parts of the touchscreen than the black dot. 

### 2.3. Data Treatment and Statistical Analysis

We applied the following criteria in RT-data cleaning: if the percentage of errors in a given participant exceeded 25%, in any of the tasks *T*1–*T*3, then all his results were excluded from further analysis. All RTs for incorrect responses were delated, as well as RTs below 50 ms, which might be considered as unintended or premature responses. Since the analysis of tasks *T*1–*T*3 parametric indices of performance speed and variability (M, iSD) considered correction for manual speed, in any case when such correction generated negative numbers the whole series of RT-data was also excluded from further computations. Due to the fact that our study assumes especially the assessment of RT-variability, also in a form of overly long responses, no cut-offs were applied to such prolonged RTs, however, based on Ulrich and Miller suggestions [41], two slowest RTs for each of tasks *T*1–*T*3 data sets were excluded.

After described data-treatment procedures, a compliance of RTs with normal distribution was analyzed for all four tasks separately with application of Kolmogorov-Smirnov test with Lilliefors correction (K-S test). Additionally, using descriptive statistics formula from STATISTICA package the level of distribution skewness had been computed. RT-s distributions has been assigned as significantly deviating from normal if the *p* level of K-S test was lower than 0.05. For the analysis of parametric indices of speed and variability (M, iSD, CoV), these variables which did not meet the assumption of compliance with the normal distribution were z-standardized to enable the application of parametric statistics in computing main effects including all three main tasks (*T*1–*T*3). In all main analyses indicators of performance speed and variability were set as dependent variables, while the level of RSI’s length and irregularity as an independent within-subjects factor. To compute relationships between these variables a repeated-measures ANOVA has been applied with Greenhouse-Geisser degrees of freedom corrections regarding effects significance level in case if the assumptions about the sphericity of covariation would be violated. Moreover, if the analysis included comparisons between more than two dependent variables, the Bonferroni correction for multiple testing was included to set the level of significance. We used Tukey’s test in post-hoc analyses and partial eta squared (*η_p_*^2^) as an effect size indicator. Analogously, repeated-measures ANOVA was used to verify whether the level of performance speed and variability was relatively stable during tasks execution, by computing mRT and iSDRT for the first and the second halves of the tasks. To compare men and women regarding performance speed and variability outcomes, a paired *t*-test was used, or, if these subgroups differed significantly in terms of age and level of education, then, to compare them, an ANCOVA would be used, with age and years of education included as controlled covariates. 

The ex-Gaussian distribution was fitted to raw RTs sets characterized by a positive skewness of dispersions significantly deviating from the normal. The ex-Gaussian distribution being a convolution of the Gaussian and exponential distributions enables to compute three independent markers: *μ*, which is a mean of the Gaussian component, *σ*, an indicator of the symmetrical, Gaussian standard deviation and *τ* covering the mean and the standard deviation of the exponential component. In general, among the ex-Gaussian parameters, the *τ* might be considered as the main indicator of IIV because it determines the proportion of the most prolonged RTs. The ex-Gaussian markers were estimated based on cleaned, but not standardized RTs according to Lacouture and Cousineau recommendations [42] with the application of the MATLAB toolbox “DISTRIB” (version: R2017a, Mathworks Inc., Natick, MA, USA). A Pearson’s *r* coefficient was used to verify for potential significant correlations between *μ* and *τ*. All time-related outcomes are given in milliseconds (ms) as means and standard deviations (±). 

This research was approved by the local medical ethics committee of the Medical University of Lublin (KE-0254/262/2020) and was carried out in compliance.

## 3. Results

From the initial sample of 135 participants, after screening them with regard to exclusion criteria, results of 16 were rejected from the entire pool due to the fact that: 6 of them had an unclear family history of psychiatric disorders, 4 appeared to be ambidextrous or had undetermined laterality for the dominant hand, 3 participants admitted having learning difficulties while attending to the primary school, 2 admitted consuming more than one energy drink before the assessment, 1 participant admitted taking benzodiazepines.

After applying data treatment rules, from the entire pool of RTs, the results of another 12 participants were eliminated, 3 subjects lacked complete data set, 4 had the percentage of errors higher than 25%, in case of another 3 participants the correction for manual speed generated negative numbers for iSD in one task and the RTs series of 2 subjects were characterized by a standout number of empty boxes after data deletion according to other criteria, therefore we decided to remove them due to the possibly increased tendency to generate random, though not explicitly erroneous reactions. Of note, initial analysis of cleaned RT data showed that in tasks *T*1–*T*3 first RTs were exceptionally long and most often met the criteria of the two slowest RTs. 

In sum, full data of 107 individuals have undergone ultimate analysis. This group contained 56 woman (52.33%) and 51 men (47.66%) with mean age of 22.75 ± 2.24 years and 16.14 ± 2.02 years of education. Men and women had relatively similar age and level of education (*p* > 0.3). 

### 3.1. RT-Data Distributional Properties and Within-Tasks Changes in Speed and Variability

According to distributional analyses for four tasks presented in Table 1, RT-data from FT and *T*1 fitted to normal distribution, while a set of RTs from tasks *T*2 and *T*3 significantly deviated from the Gaussian dispersion. The level of skewness was positive and increased for the four consecutive tasks reaching the highest value for task *T*3.

Repeated-measures ANOVA for mRT and iSDRT data from the first and second halves of three separately analyzed tasks (*T*1, *T*2, *T*3), confirmed that in *T*1 subtest mRT was significantly higher in the first half than in the second one (336.34 ms versus 282.29 ms): F(1, 103) = 91.87, *p* < 0.0001, *η_p_*^2^ = 0.47, additionally, iSD was higher in the first comparing with the second half of task (52.12 ms versus 40.52 ms), but in this case the difference reached the level of statistical trend: F(1, 103) = 2.98, *p* = 0.078, *η_p_*^2^ = 0.03. Analogous analyses conducted for FT, *T*2 and *T*3 tests showed that there were no significant differences regarding mRT and iSDRT for the first and the second halves of tests (all *p* > 0.1). 

### 3.2. RSIs-Related Within-Subjects Effects 

There was a significant effect regarding the overall number of errors committed in three consecutive tasks: F(2, 192) = 4.41, *p* = 0.011, *η_p_*^2^ = 0.045, consisting in a significantly higher number of errors observed in task *T*3 comparing with tasks *T*1 and *T*2 (post-hoc Tukey tests *p* < 0.012). 

**Figure 1:**
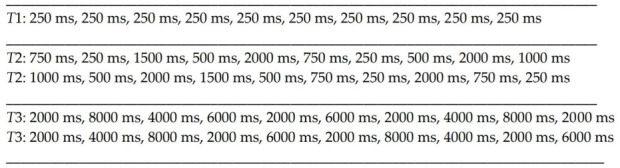
Examples of the distribution of ten consecutive RSIs in three main tasks. The *T*1 always has the same short and regular interval of 250 ms. In task *T*2, individual intervals are variable, but they fall within the range between 250 and 2000 ms, the longest RSIs occurs in 20% of all cases. As shown in the two examples, although the length of successive intervals was different as a result of computer-moderated randomization, the sum of the duration of all RSIs in the task is always the same, in both cases, it was 9500 ms. Similarly, two examples show the possibility of distributing consecutive intervals in the *T*3 task, in which the RSI segments range from 2000 to 8000 ms. As in task *T*2, the successive RSIs were different, but the sum of all of them in both examples is the same, 44,000 ms. Partial randomization of the distributions of individual intervals was performed so that any fluctuations in reaction times associated with IIV did not result from the fact that the longest intervals, which according to theoretical assumptions should generate the largest range of IIV, always occurred in the same parts of the test.

Descriptive statistics for the FT test were as follows: M = 160.91 ms ± 17.01, iSD = 22.59 ms ± 16.32, CoV = 0.14 ± 0.13. As for mRT in all three tasks, corrected for the mean time of FT task, repeated-measures ANOVA revealed that mRT in each subsequent task was significantly longer than the previous one: F(2, 206) = 1179.6, *p* < 0.0001, *η_p_*^2^ = 0.92 (all post-hoc tests *p* < 0.001) (details in Table 2). Repeated measures ANOVA regarding individual standard deviations (iSD) for tasks *T*1, *T*2 and *T*3, corrected for iSD from FT [F(2, 206) = 108.11, *p* < 0.0001, *η_p_*^2^ = 0.52] confirmed that corrected iSD for the third task was significantly higher than for *T*1 and *T*2 (post-hoc test *p* < 0.001), however, difference between iSD in *T*1 and *T*2 reached level of statistical trend, considering Bonferroni correction for multiple tests (*p* = 0.033). The effect of task on RT coefficient of variation (CoV), corrected for CoV of the FT task was also significant: F(2, 206) = 16.56, *p* < 0.0001, *η_p_*^2^ = 0.14. CoV of *T*3 was the highest, followed by *T*1 and *T*2, post-hoc analysis revealed, that significant differences concerned pairs *T*2 and *T*3 (*p* < 0.0001) and *T*1 and *T*2 (*p* < 0.002), however the difference between CoV of *T*1 and *T*3 was not significant (*p* = 0.185) (Figure 1).

**Figure 2:**
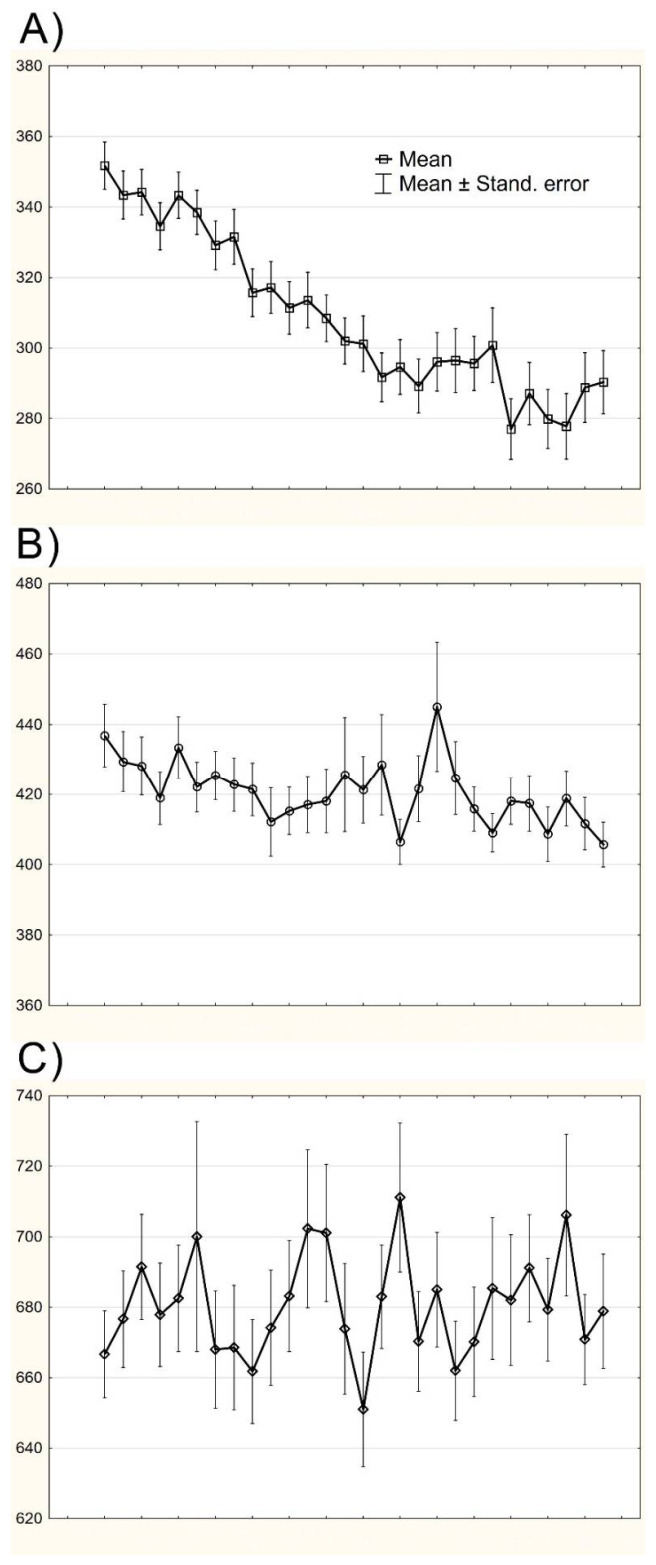
The range of trial-to-trial variability for FT-uncorrected series of consecutive RTs in (**A**) task *T*1, (**B**), *T*2 and (**C**) *T*3 performed by the whole analyzed sample. Figure shows averaged mRTs and the range of mean ± standard error of the mean. The curve containing RTs for *T*1 (**A**) task is descending consistently with statistical analysis confirming significant, gradual performance acceleration. On the other hand, fluctuations in successive RTs are very slight. The level of RTs variability in tasks *T*2 and *T*3 is stable throughout the performance and it seems evident, that fluctuations between consecutive RTs are most pronounced in *T*3 task (**C**). Significant acceleration of performance speed in *T*1, leading to the substantial difference between RTs from the initial and final phases of execution, might have caused that parametric measures of variability (iSD and CoV) showed some similarities in this respect between the three tasks, despite a clear difference in the range of fluctuations among successive RTs, especially in tasks *T*1 and *T*3. All data are presented in milliseconds scale and concerns sets of RT after initial cleaning for the extreme outliers. Axis x—a set of consecutive RTs, axis y—time in milliseconds.

**Figure 3:**
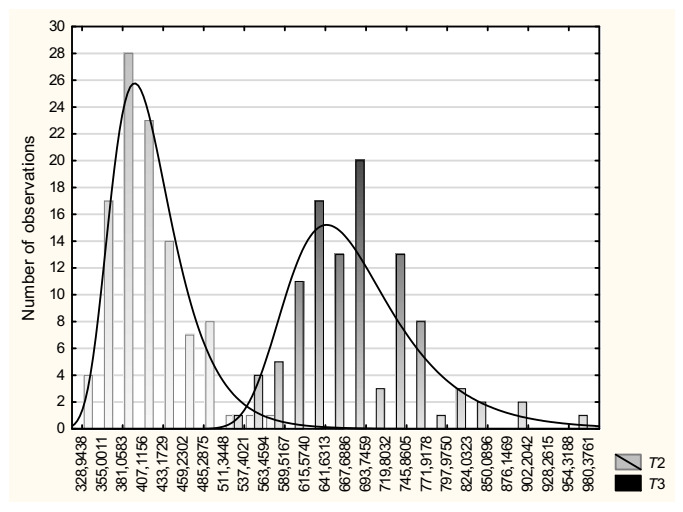
Distributions of RTs from tasks *T*2 and T3. The histograms show the fitness of the empirical data to the ex-Gaussian curves, with increased mean RTs and right-side exponential tail in task *T*3 comparing with *T*2. Axis x—time in milliseconds, axis y—number of observations (See the Supplementary Materials containing the other visualization of RTs-related data from tasks *T*1, *T*2 and *T*3 [43,44]).

As it might be inferred from the distribution skewness of tasks *T*2 and *T*3, their RT-data fitted the ex-Gaussian dispersion properties with elongated right-side convolution tail, as depicted in Figure 2. Repeated measures ANOVAs confirmed that all three ex-Gaussian parameters were significantly higher for *T*3 comparing with *T*2 tests. In details, the results were as follows: regarding *μ*: F(1, 105) = 876.76, *p* < 0.0001, *η_p_*^2^ = 0.89, regarding *σ*: F(1, 105) = 77.81, *p* < 0.0001, *η_p_*^2^ = 0.43 and regarding *τ*: F(1, 105) = 62.14, *p* < 0.0001, *η_p_*^2^ = 0.37 (Table 2, Figure 3). There were no significant correlations between *μ* and *τ* parameters within *T*2 or *T*3 tasks (all *p* > 0.3).

### 3.3. Sex Differences in Performance Speed and Variability

Comparisons of male and female participants in terms of variables describing performance speed and IIV revealed that: women were significantly slower in FT test (164.27 ms ± 13.54 versus 156.99 ms ± 19.75 in men, t(105) = 2.21, *p* = 0.028) and had higher indicators of variability in *T*1 task: iSD = 33.89 ± 29.47 in women and 17.61 ± 25.16 in men, t(105) = 3.00, *p* = 0.003, CoV = 0.34 ± 0.45 in women and 0.16 ± 0.25 in men subgroups, t(105) = 2.48, *p* = 0.014.

## 4. Discussion

The main goals of our study were to construct a cognitive assessment method purposefully eliciting controlled amount of RT-related trial-to-trial variability and to verify the principal assumption, according to which this effect might be induced by manipulating the length and regularity of intervals between previous reaction and the onset of the next stimuli (RSIs). We developed a digital method containing four subtests including one measuring manual speed and its fluctuations, and three with incremental level of RSI length and within-task variability. It was assumed that the performance of the task with the longest and most irregular RSIs (subtest *T*3) will be characterized by the highest amount of IIV also when the mean RT (mRT) and manual speed and variability will be controlled. In general, obtained results seem to confirm these hypotheses. We started analyses by evaluating the skewness of RTs distributions and showed that in all tasks the skewness was positive and gradually increased with the range of RSIs manipulation. This distributional measure reached the largest range for the last task (*T*3). It is also worth noticing, that the dispersion of the RTs series became non-compliant with the Gaussian distribution in task *T*2, which is the first subtest with variable RSI level. Increasing positive skewness encompasses gradual enlargement of RTs outliers, which is in line with the general features of RT-related IIV, consisting of the buildup of substantially elongated and not shortened RTs [45].

Significant effect of RSI manipulations on processing consistency was also corroborated by psychometric and distributional, ex-Gaussian indicators, although with one important exception. All applied measures of trial-to-trial variability, such as iSDRT, CoV, *σ* and *τ* were the highest for *T*3 task and these results fully corroborates our expectations. However, in the case of the coefficient of variation, there was no significant difference between *T*3 and *T*1 tasks, what is contrary to hypotheses and, above all, to the features of the RTs distributions skewness discussed above, since the skewness of *T*3 task was fifteen times greater than in the *T*1 (2.629 versus 0.174). As depicted in Figure 1. and proved by the statistical analysis of within-task changes in mRT and iSDRT, the performance of *T*1 characterized gradual acceleration and a decrease in processing fluctuation. Progressive reduction of successive RTs and the scope of its variability causes inaccurate computation of psychometric measures of IIV, because the comparison of RTs from the beginning and the end of its series will generate outcomes falsely suggesting substantial dissimilarities between the shortest and the longest RTs. On the other hand, the presence of performance acceleration in the *T*1 task was very likely considering the fact, that RSI in this subtest is very short (250 ms) and above all, fixed intervals, which enabled the development of an anticipatory response being a result of implicit learning. It was proved numerous times, that in almost all version of RT-tests in which stimuli are displayed regularly, and the regularity might apply both the temporal and spatial aspects of stimuli presentations, the intra-task practice effect will occur due to involvement of implicit learning causing gradual decrease of RTs [46,47,48]. Despite the fact that the acceleration of the *T*1 performance can be easily explained by referring to mentioned classic research, the effects of this acceleration on the IIV measures clearly indicate the necessity to control for actual RTs sequences in terms of its global changes ongoing over time of performance. As in the case of an SRT study with healthy participants, when an RTs acceleration should be expected, in studies involving neuropsychiatric patients, the opposite effect may occur, consisting of gradual lengthening of successive RTs, accompanied by increased processing fluctuations arising from cognitive fatigability [49,50,51]. Considering the above, in order to correctly estimate IIV, it is necessary to control for the potential acceleration or deceleration occurring during task performance. 

As for our results, taking into account only the comparison between the *T*2 and *T*3, i.e., those tasks in which the range of fluctuations between adjacent RTs were stable on the scale of the entire task, all the IIV measures were significantly higher for *T*3 than *T*2, also when the motoric speed and variability were taken into account. Remembering that developed measuring tool was design to assess neuropsychiatric patients, we believe that the control of motor speed is critical. Motoric slowdown, including decreased manual speed, is a typical feature of disorders such as schizophrenia and bipolar disorder [52,53], therefore the comparison of the aforementioned clinical groups and healthy subjects in terms of any RT-data, without taking into account manual slowing can potentially lead to misleading conclusions. 

As hypothesized, the greatest range of RTs fluctuations concerned the task with the most variable and the longest RSIs. Our assumption linking manipulation of RSI with IIV was based on the clinically-driven Stuss and co-workers model of attentional-executive system associated with activity of the frontal lobes [54] and, on the other hand, on the neuro-energetic theory of mind wandering presented by Killen [35]. Especially Killen suspected, that mind wandering, revealing in RT-tasks as a heightened scope of VII, might particularly dominate the performance of low-paced tasks, since attentional-energetic resources cannot be constantly expended without external stimulation and such stimulation is provided by fast-paced tasks. In fact, when analyzing low-paced tests from the point of view of its temporal organization, the long-lasting RSIs make this task consisting primarily of waiting for a stimulus. In other words, having in mind average RTs in this task and the length of RSIs, a subject performing the *T*3 subtest spends much more time on not reacting than reacting. Such characteristics of this subtest implies another, even more contemporary, neuroscientific explanation of the relationship between long RSIs and IIV, which might be referred to the phenomena of functional competition between on-task and off-task neural networks. Weissman et al. [55], in their seminal paper disclosing neural underpinning of momentary attentional lapses indicated by the prolonged RTs occurring during selective attention task performance, revealed that lapses arise when activation of brain regions such as anterior cingulate gyrus, middle frontal gyrus and right inferior frontal gyrus, granting executive and attentional control over receptive structures is diminished, but slips of attention were also associated with insufficient deactivation of structures constituting the posterior hub of the Default Mode Network (DMN). The negative impact of spontaneous overactivation or hyperconnectivity within DMN on cognitive processing was also documented in clinical research [56], of note, in our previous study on neurophysiological basis of impaired cognitive initiation in schizophrenia we have also reported that transition between off-state to task-state in these patients was inhibited by hyper-synchronizations comprising neural structures forming the posterior hub of the DMN (posterior cingulate gyrus, cuneus and precuneus) [57]. Even though this assumption undoubtedly requires empirical verification, we postulate that heightened amount of elongated RTs appearing in tasks which performance is associated with substantial proportion of phases not requiring any activity, might be secondary to the periodic inclusions of the DMN network during the performance of such tasks and reorientation of attention resources from external stimuli to internal mentation. In other words, the extent to which an RTs series taken from the low-paced task is interspersed by substantially prolonged RTs might be potentially sensitive to individual differences in the strength of anti-correlation between off-task and on-task networks, which is one of the fundamental requirements for efficient cognitive processing [58]. Even though the presented interpretation of obtained results seems justified, relationships between RSIs’ length and regularity and mRTs and their variability might be even more complex, considering that task T3 was characterized simultaneously by prolonged, but also irregular intervals. In fact, there is quite a long list of factors influencing relationships between RSI or foreperiods and distributional properties of RTs series [37,59], and it is fully justified to undertake further studies which would more directly answer the question, whether the length or irregularity of RSIs affects RTs variability to the most extent. 

Despite achieved research goals and corroboration of proposed hypotheses, our study has some limitations which should be addressed. The presented assessment tool is still purely experimental, and before taking further steps necessary to form it as a diagnostic method that can be used in research and clinical practice, it must undergo a more typical psychometric process evaluating its validity and reliability. In addition, it might be necessary to further refine various technical solutions contributing to more complete control of potential confounding factors, for example, the exact position of the finger with which the subjects perform tasks during the phase of waiting for the stimulus, for example, in a way it was solved in the Cambridge Neuropsychological Test Automated Battery (CANTAB) [60]. Controlling for the finger position might be of special importance especially when applying tasks in clinical populations. 

As it was repeatedly noted, construction of the method assumed its use in research involving clinical groups such as neuropsychiatric patients, so, it is obvious that the next step is to examine such groups, analyze the suitability of the adopted technical solutions to specificity of such patients and to demonstrate significant differences between them and the control group in IIV measures. As for the relationships between RSI length and regularity and the range of IIV, although in our opinion such a relationship has been demonstrated, the calculation of IIV measures must always take into account that an increase in RSIs irregularity entails an increase in mRT, therefore it necessary to show that any intensification in IIV in clinical groups is not just an effect elongation of mRTs. Moreover, in studies of any clinical sample, due to the possibility of increased fatigability, it is necessary to control the potential influence of the sequence of tasks on the IIV measures, so, at the level of the group, the order of the tasks should be counterbalanced across participants. In the case of comparing the results of the applied tasks in clinical and in healthy controls groups, the sequence of tasks should, however, be the same to control for the potential effect of the tasks’ order on the obtained parameters of RTs variability. 

## 5. Conclusions

According to our knowledge, we have developed one of the very few methods intentionally constructed to elicit and analyze the fluctuations in the RTs series. The structure of tasks was based on well-documented theoretical premises. We included the control of many potential confounding variables, such as manual speed, potential intra-task changes of the overall speed and variability and multiple levels of the extent of the impact of RSI traits on IIV. Above all, we proposed very simple tasks which is not principally based on higher-order, complex cognitive functions, but can generate the expected IIV level in a controlled manner.

## Figures and Tables

**Table 1:** Compliance with the normal distribution and the level of skewness in sets of RT-data in four consecutive tasks.

Task	Kolmogorov-Smirnov Test with Lilliefors Correction *p*-Values	Distribution Skewness	Classification of Distribution’s Normality
FT	>0.2	0.109	normal
*T*1	>0.2	0.174	normal
*T*2	<0.01	0.824	non-normal
*T*3	<0.001	2.629	non-normal

**Table 2:** Results of speed and variability indicators in three main tasks with repeated-measures ANOVA outcomes. Parametric indices (mRT, iSD, CoV) were corrected for the manual speed and variability assessed by the *FT* test. The ex-Gaussian (*μ*, *σ*, *τ*) indexes were calculated on non-normalized RTs without FT-correction, however after initial data treatment, as presented in Section 2.3.

	*T*1 M (SD)	*T*2 M (SD)	*T*3 M (SD)	F	*p*	*η_p_* ^2^	Post Hoc
mRT	148.03 (61.26)	256.87 (42.48)	517.37 (72.04)	1179.6	<0.0001	0.92	*T*1 < *T*2 < *T*3
iSD	26.38 (31.18)	35.07 (42.08)	109.33 (70.63)	108.11	<0.0001	0.52	*T*1, *T*2 < *T*3
CoV	0.17 (0.10	0.13 (0.07)	0.19 (0.07)	16.56	<0.0001	0.14	*T*2 < *T*1, *T*3
mu (*μ*)	-	347.39 (45.59)	584.94 (86.46)	876.76	<0.0001	0.89	*T*2 < *T*3
sigma (*σ*)	-	16.03 (14.17)	41.98 (27.61)	77.81	<0.0001	0.43	*T*2 < *T*3
tau (*τ*)	-	63.34 (44.58)	114.80 (67.91)	62.14	<0.0001	0.37	*T*2 < *T*3

Note. All data presented in milliseconds, *η_p_*^2^*—*effect size indicator, post hoc tests were performed using Tukey’s test with Bonferroni correction for multiple comparisons.

## Data Availability

The data presented in this study are available on request from the corresponding author. The data are not publicly available due to lack of institutional online database.

## Supplementary Materials

The following are available online at https://www.mdpi.com/article/10.3390/brainsci11050669/s1, Figure S1: The probability-probability plot showing the range of fit and mismatch to the theoretical distribution of RTs-data from tasks *T*1, *T*2 and *T*3. Empirical cumulatives indicate the range of deviations from expected normal distribution.
