# Supplementary material for: Manipulations of the Response-Stimulus Intervals as a Factor Inducing Controlled Amount of Reaction Time Intra-Individual Variability
Source: Brain Sci. 2021 May 20;11(5):669. doi: 10.3390/brainsci11050669 (PMC8161342; doi:10.3390/brainsci11050669)
Supplement: Supplementary file 1 [file brainsci-11-00669-s001.zip › brainsci-1176800-supplementary.pdf]

brain sciences

Supplementary Material

Manipulations of the response-stimulus intervals as a factor inducing controlled amount of reaction time intra-individual variability

Paweł Krukow, Małgorzata Plechawska-Wójcik and Arkadiusz Podkowiński

Citation: Krukow, P.; Plechawska-Wójcik, M.; Podkowiński, A. Manipulations of the Response-Stimulus Intervals as a Factor Inducing Controlled Amount of Reaction Time Intra-Individual Variability. *Brain Sci.* **2021**, *11*, 669. https://doi.org/10.3390/brainsci11050669

Academic Editors: Marco Bove and Laila Craighero

Received: 24 March 2021
Accepted: 18 May 2021
Published: 20 May 2021

Copyright: © 2021 by the authors. Licensee MDPI, Basel, Switzerland. This article is an open access article distributed under the terms and conditions of the Creative Commons Attribution (CC BY) license (http://creativecommons.org/licenses/by/4.0/).

The analysis and visual representation of the specific characteristics of the distribution of the reaction time series, especially those differing to varying degrees from the normal distribution, may be performed using various computational approaches. Some authors [43,44] suggest to visualize such data based on the percentile distributive function, or/and to use cumulative distributive functions (CDF) to see the skewness of the variables in a percentile based function. We decided to add Figure S1. showing the so-called 'probability-probability plot '(also known as a P-P plot), enabling to visualize and evaluate the fit of the theoretical distribution (here it is Gaussian dispersion) to the empirical distribution of the data. In probability-probability plots (or P-P for short), the empirical cumulative distribution function is plotted against the theoretical cumulative distribution function.

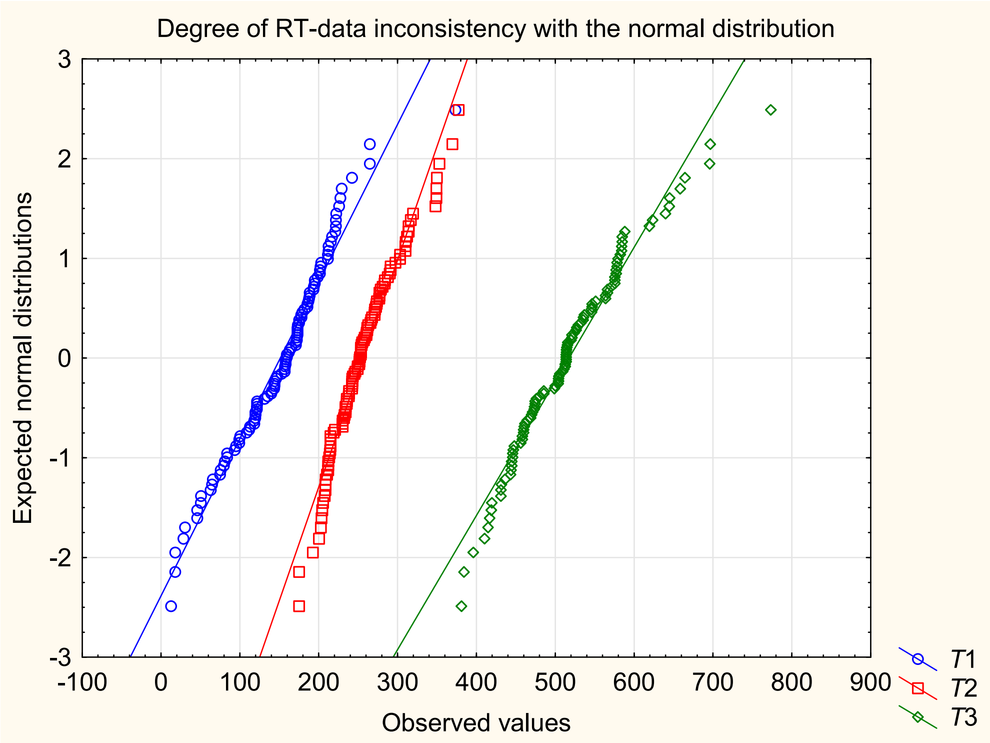

Figure S1. The probability-probability plot showing the range of fit and mismatch to the theoretical distribution of RTs-data from tasks *T*1, *T*2 and *T*3. Empirical cumulatives indicate the range of deviations from expected normal distribution.
